# Expression of long noncoding RNAs in the ovarian granulosa cells of women with diminished ovarian reserve using high-throughput sequencing

**DOI:** 10.1186/s13048-022-01053-6

**Published:** 2022-10-29

**Authors:** Li Dong, Xin Xin, Hsun-Ming Chang, Peter C. K. Leung, Chen Yu, Fang Lian, Haicui Wu

**Affiliations:** 1 First School of Clinical Medicine, Shandong University of Traditional Chinese Medicine, Jinan, 250011 China; 2 Department of Obstetrics and Gynaecology, BC Children’s Hospital Research Institute, University of British Columbia, Vancouver, British Columbia Canada; 3 MedicineReproductive and Genetic Center of Integrated Traditional and Western Medicine, the Affiliated Hospital of Shandong University of Traditional Chinese Medicine, Jinan, 250014 China

**Keywords:** Diminished ovary reserve, Ovarian granulosa cells, Long non-coding RNA, High-throughput sequencing

## Abstract

**Background:**

Infertility is a global reproductive-health problem, and diminished ovarian reserve (DOR) is one of the common causes of female infertility. Long noncoding RNAs (lncRNAs) are crucial regulators of numerous physiological and pathological processes in humans. However, whether lncRNAs are involved in the development of DOR remains to be elucidated.

**Methods:**

Ovarian granulosa cells (OGCs) extracted from infertile women with DOR and from women with normal ovarian reserve (NOR) were subjected to high-throughput sequencing. Comprehensive bioinformatics analysis was conducted to identify the differential expression of messenger RNAs (mRNAs) and lncRNAs. Sequencing results were validated by the selection of lncRNAs and mRNAs using real-time reverse transcription-quantitative polymerase chain reaction (RT-qPCR).

**Results:**

Compared with the NOR group, a total of 244 lncRNAs were upregulated (53 known and 191 novel), and 222 lncRNAs were downregulated (36 known and 186 novel) in the DOR group. Similarly, 457 mRNAs had differential expression between the two groups. Of these, 169 were upregulated and 288 were downregulated. Bioinformatics analysis revealed that the differentially expressed genes of mRNA and lncRNAs were considerably enriched in “cell adhesion and apoptosis”, “steroid biosynthesis”, and “immune system”. A co-expression network comprising lncRNAs and their predicted target genes revealed the possible involvement of the “thyroid hormone signaling pathway” and “protein binding, digestion and absorption” in DOR pathogenesis. The expression of SLC16A10 was positively regulated by multiple lncRNAs. After RT-qPCR validation of seven differentially expressed lncRNAs and mRNAs, respectively, the expression of lncRNA NEAT1, GNG12, ZEB2-AS1, and mRNA FN1, HAS3, RGS4, SUOX were in accordance with RNA-sequencing.

**Conclusions:**

We presented the first data showing that the expression profiles of lncRNA and mRNA in OGCs between NOR and DOR patients using RNA sequencing. The lncRNAs and mRNAs that we identified may serve as novel diagnostic biomarkers for patients with DOR.

**Supplementary Information:**

The online version contains supplementary material available at 10.1186/s13048-022-01053-6.

## Background

Infertility is a worldwide problem involving couples of reproductive ages. It will become the third major health threat after cancer and cardiovascular diseases [1]. As a serious medical problem, infertility can have destructive effects on the quality of life and marital satisfaction. As economic and social pressures increase, the age at which women have children continues to increase. The term of diminished ovarian reserve (DOR) was first described by Navot and colleagues in 1987 [2]. It is characterized by a decline in the quantity and/or quality of oocytes, with increased serum levels of follicle-stimulating hormone (FSH) and decreased serum levels of anti-Müllerian hormone (AMH) [3]. The main clinical manifestations are irregular menstruation, perimenopause syndrome, and infertility. The United States Assisted Reproductive Technology Population Survey revealed that the prevalence of DOR has increased from 19% in 2004 up to 26% in 2011 [3], and is gradually increasing as a proportion of infertile women. Studies have demonstrated that DOR typically develops into premature ovarian failure (POF) in 1–6 years [4].

Women with DOR usually result in poor pregnancy outcomes and present major challenges in reproductive medicine, particularly receiving in vitro fertilization/intracytoplasmic sperm injection (IVF/ICSI) treatment [5]. However, the etiology and pathogenesis of DOR are not clear. The key etiological factors are age, genetics, iatrogenicity (surgery, radiotherapy, or chemotherapy), and immune and environmental factors [6]. Therefore, to improve pregnancy outcomes in women with DOR, the clinicians should focus on the molecular mechanisms underlying DOR occurrence. In this way, timely prediction of the diagnosis, necessary interventions, and therapeutic measures through identification of clinical biomarkers can be undertaken.

Long noncoding RNAs (lncRNAs) are functional RNA molecules of > 200 nucleotides in length that cannot be translated into proteins. Recently, studies have revealed that lncRNAs regulate the expression of genes in terms of transcriptional, posttranscriptional, and epigenetic modifications [7]. These actions may affect the growth, development, aging, and disease progression of organisms [8]. Additionally, lncRNAs are involved in the regulation of stem cell differentiation, embryonic development, cell proliferation, apoptosis, cell metabolism, and immune reaction [9, 10]. Recent studies have shown differential expression of lncRNAs in several gynecological disorders, such as endometriosis [11], premature ovarian insufficiency (POI) [12], and polycystic ovary syndrome (PCOS) [13]. Although several lncRNAs have been found to be involved in the abovementioned ovarian diseases, the association between lncRNAs and DOR is largely unknown.

Technologies based on transcription, gene chips, and high-throughput sequencing, have found that various lncRNAs are related to the occurrence, development, and treatment of diseases [14]. The use of biological methods to study differences in the expression of lncRNAs between patients and healthy individuals and prescreening lncRNAs by high-throughput sequencing may aid in the discovery of new targets related to many diseases. In-depth study of the biomolecular mechanisms of lncRNAs can help explain the complexity and pathological states of diseases and support the diagnosis and treatment of refractory diseases.

In this study, we aimed to employ transcriptome analysis to investigate the biological role of lncRNAs during DOR progression. Ovarian granulosa cells (OGCs) from women with DOR and those from women with normal ovarian reserve (NOR) were obtained to perform RNA-sequencing (RNA-seq) and analyze the expression profiles between two groups. Bioinformatics analysis was undertaken to identify differentially expressed (DE) lncRNAs and messenger (m) RNAs and their associated functions and signaling pathways. In addition, we applied real-time reverse transcription-quantitative polymerase chain reaction (RT–qPCR) to verify the differential expression of seven lncRNAs and mRNAs. These aberrantly expressed lncRNAs and mRNAs may serve as potential biomarkers for the development of DOR and may introduce new routes for the diagnosis of women with POF.

## Results

### Baseline characteristics of patients used for sequencing

As presented in Table 1, patients in the DOR group had low AMH levels, low antral follicle count (AFC), low number of oocytes obtained and high basal FSH levels compared to the NOR group, the differences in the above characteristics being indicators of ovarian reserve (*P* < 0.001). No other features were significantly different (*P* > 0.05).

**Table 1:** Comparison of the baseline characteristics between the NOR group and DOR group used for sequencing

	NOR (n = 6)	DOR (***n*** = 6)	P
Patient age (years)	30.50 ± 1.76	35.17 ± 4.88	0.068
Infertility duration (years)	4.50 ± 2.95	5.17 ± 2.40	0.677
Infertility type (%)			0.567
Primary	4/6 (66.7)	2/6 (33.3)	
Secondary	2/6 (33.3)	4/6 (66.6)	
BMI (kg/m^2^)	22.72 ± 0.98	21.75 ± 1.33	0.181
AMH (ng/mL)	4.64 ± 1.11	0.79 ± 0.23	< 0.001
Basal FSH (mIU/mL)	6.99 ± 1.40	13.57 ± 2.04	< 0.001
AFC (n)	25.67 ± 2.73	6.83 ± 1.94	< 0.001
Previous IVF/ICSI attempts (n)	0.00 ± 0.00	1.17 ± 1.60	0.135
Number of oocytes retrieved	16.17 ± 3.49	4.00 ± 2.28	< 0.001

Data are the mean ± SD and proportion (%)

*AMH* Anti-Müllerian hormone, *AFC* Antral follicle count, *BMI* Body mass index, *FSH* Follicle-stimulating hormone, *ICSI* Intracytoplasmic sperm injection, *IVF* In vitro fertilization

### Sequencing of complementary (c) DNA libraries and transcriptome profiles of OGCs

A total of 999,067,624 raw reads were acquired using the NovaSeq™ 6000 platform (Illumina, San Diego, CA, USA). After excluding adapter contamination, undetermined bases, and low-quality bases, 902,773,866 clean reads were obtained, accounting for 135.42 GB. Although the GC content of the clean data was ~ 42%, the quality scores of clean reads were > 99.9 and > 98.83% for Q20 and Q30, respectively. In summary, these results indicated that the reliability and quality of the sequencing data were sufficient for further analyses (Table 2). Over 96% of clean reads were mapped to the reference human genome using HISAT (http://daehwankimlab.github.io/hisat2/), including uniquely mapped reads from 84.10 to 86.76% (Supplemental Table 1).

**Table 2:** Statistical data of the reads for 12 cDNA libraries

Sample	Raw reads	Raw bases	Clean reads	Clean bases	Valid ratio (reads)	Q20%	Q30%	GC content (%)
DOR_1	95,746,384	14.36G	87,242,788	13.09G	91.12	99.99	98.96	42
DOR_2	90,791,244	13.62G	79,121,838	11.87G	87.15	99.99	98.88	43
DOR_3	72,114,510	10.82G	66,912,764	10.04G	92.79	99.99	98.96	41.50
DOR_4	70,676,974	10.60G	65,727,486	9.86G	93.00	99.99	98.97	41
DOR_5	91,948,332	13.79G	85,217,766	12.78G	92.68	99.99	98.93	42
DOR_6	81,704,846	12.26G	74,061,172	11.11G	90.64	99.99	98.83	44
NOR_1	88,597,962	13.29G	81,949,398	12.29G	92.50	99.99	98.91	42
NOR_2	67,940,108	10.19G	62,960,406	9.44G	92.67	99.99	98.93	41.50
NOR_3	66,981,682	10.05G	61,871,236	9.28G	92.37	99.99	98.98	42
NOR_4	88,814,944	13.32G	73,435,164	11.02G	82.68	99.99	98.91	43.50
NOR_5	90,275,194	13.54G	77,710,494	11.66G	86.08	99.99	98.88	43
NOR_6	93,475,444	14.02G	86,563,354	12.98G	92.61	99.99	98.97	42

### Identification of lncRNAs and mRNAs

**Fig. 1:**
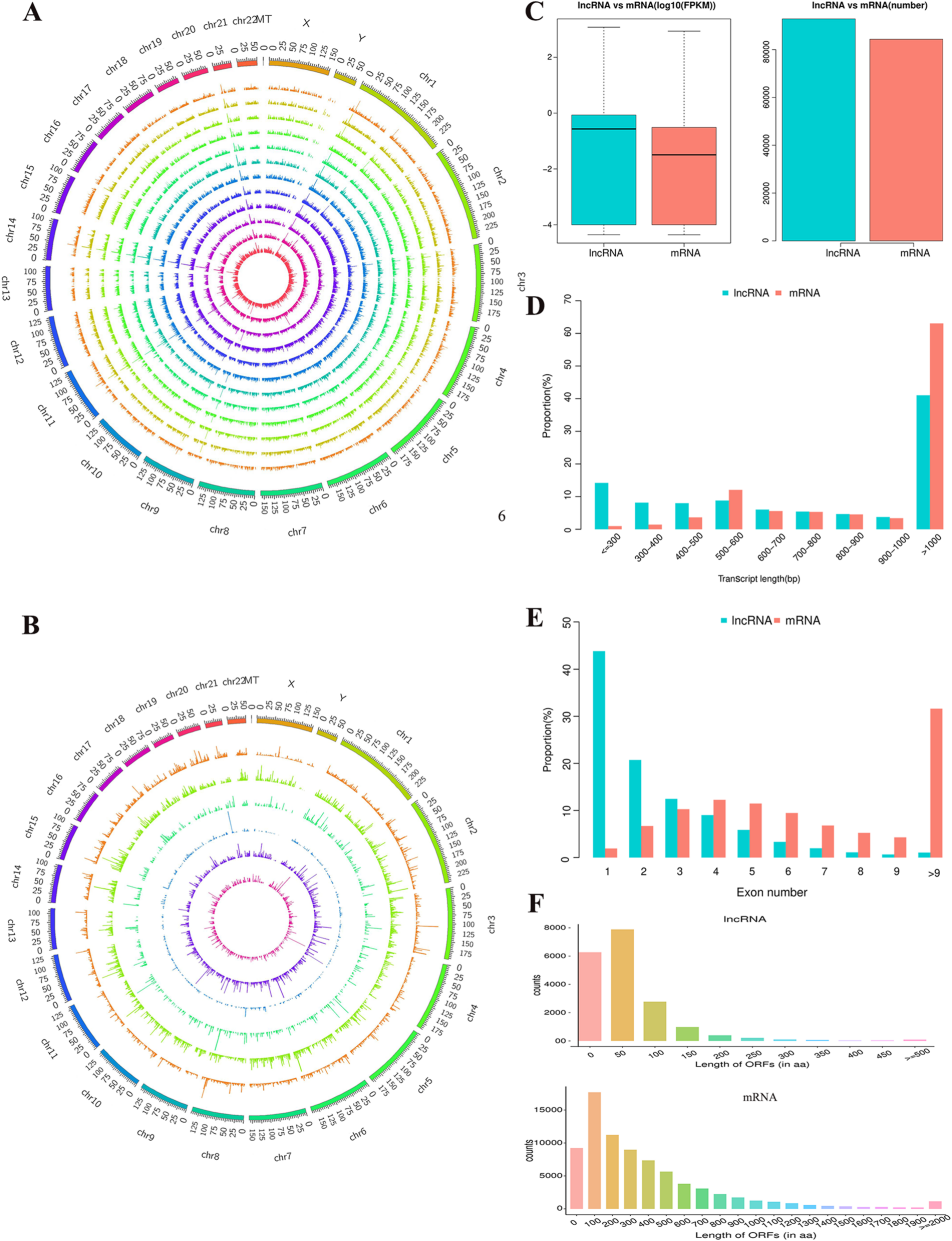
Comparison of the properties of mRNA and lncRNA from women with NOR or DOR. **a** Genomic mapping of lncRNAs in different samples. **b** Location of lncRNA types on 46 chromosomes. Each circle represents a type of lncRNA and corresponds to “i”, “j”, “o”, “u”, or “x” from the outer circle to the inner circle. **c** The expression and amount of lncRNAs and mRNAs. **d** Distribution of the transcript length of lncRNAs and mRNAs. **e** Distribution of the exon number of lncRNAs and mRNAs. **f** Distribution of the ORF length of lncRNAs and mRNAs

To further recognize the protein-coding ability of unknown transcripts, Coding Potential Calculator (CPC; http://cpc2.gao-lab.org/) and Coding-Non-Coding Index (CNCI; https://github.com/www-bioinfo-org/CNCI/) were designed to exclude those with coding potential. As a result, a total of 45,333 novel lncRNAs were detected from 12 cDNA libraries (Supplemental Table 2). These lncRNAs were dispersed evenly across the 46 human chromosomes (Fig. 1a). With regard to the genomic location of the lncRNAs, the types of lncRNAs were class_code “i” (intraintronic transcript, 72.24%), class_code “u” (intergenic transcript, 18.37%), class_code “o” (sense transcript, 3.55%), class_code “j” (bidirectional transcript, 3.25%), and class_code “x” (antisense transcript, 2.59%). These lncRNAs showed no apparent bias for genomic location (Fig. 1b). Additionally, a total of 47,520 known lncRNAs were identified between the two groups.

We compared lncRNAs with mRNAs to illustrate the structural features of lncRNAs. The expression and number of lncRNAs were comparable to those of mRNAs (Fig. 1c). Furthermore, the median length of lncRNA transcripts was 1504 bp, which was shorter than the median length of 2317 bp for mRNA transcripts. These findings suggest that these lncRNAs are shorter than the mRNAs (Fig. 1d). There were fewer exons in lncRNAs (mean, 3) than in mRNAs (mean, 9). In total, 68.86% of mRNAs had ≥5 exons, whereas 77.02% of lncRNAs had ≤3 exons (Fig. 1e). Moreover, in OGCs, the predicted open reading frame (ORF) length of lncRNAs was shorter than that of mRNAs (Fig. 1f).

### Expression profile of lncRNAs

**Fig. 2:**
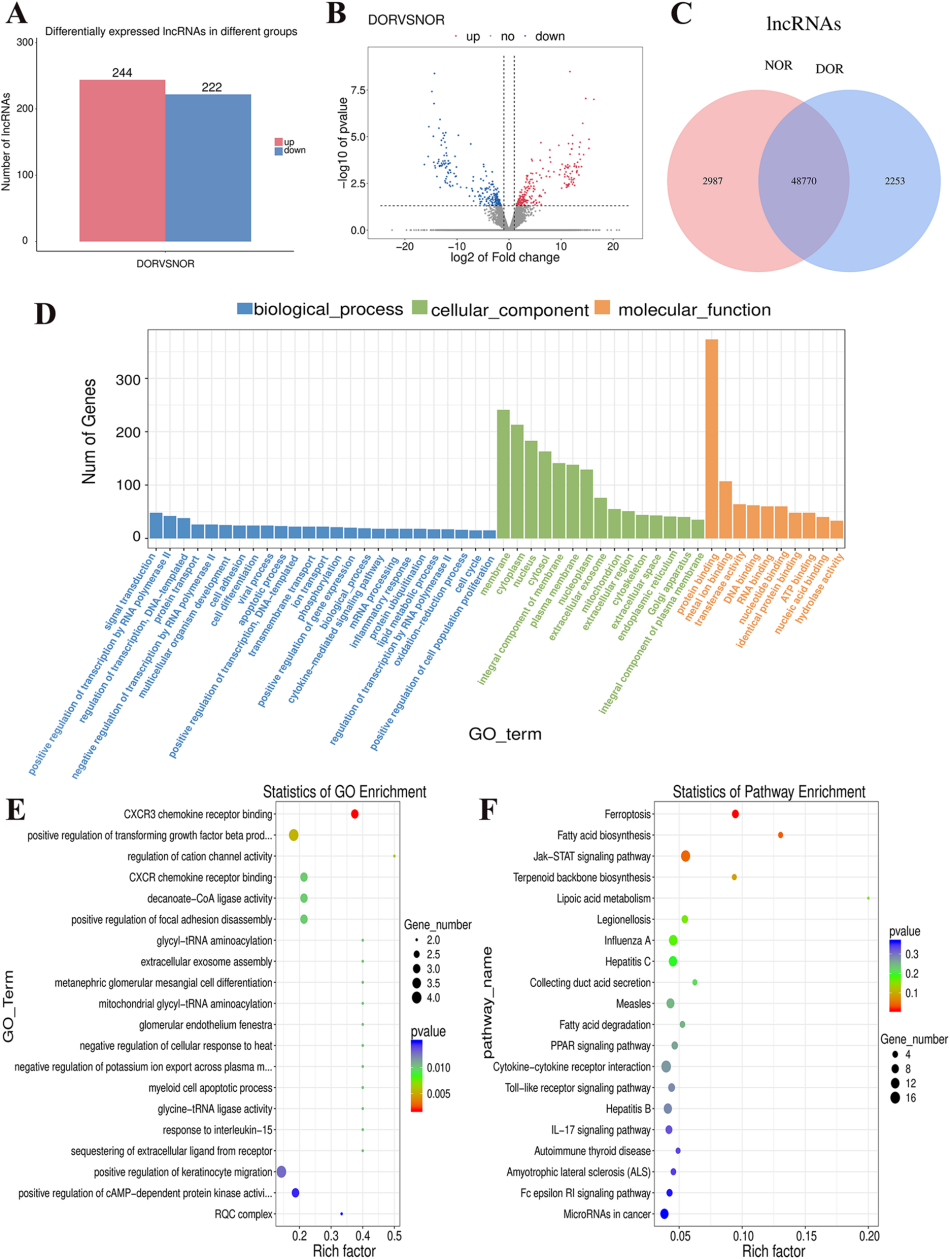
Identification and characterization of the DE lncRNAs between women in the DOR group and NOR group. **a** Number of DE lncRNAs with upregulated and downregulated expression. **b** Volcano plot of the DE lncRNAs. **c** Venn diagram showing the number of unique and common lncRNAs between the two groups. **d** Histogram of the GO-enriched terms for the target genes of the DE lncRNAs. **e** Scatter plot of the GO enrichment for the target genes of the DE lncRNAs. **f** Scatter plot of the signaling pathway enrichment for the target genes of the DE lncRNAs using the KEGG database

We discovered that 466 lncRNAs had differential expression between the DOR group and NOR group. Of these, 244 were upregulated (53 known and 191 novel), and 222 were downregulated (36 known and 186 novel) (Supplemental Table 3, Fig. 2a-b). We also calculated the average expression of lncRNAs between the NOR and DOR groups separately, filtered out genes with fragments per kilobase of exon model per million mapped reads (FPKM) values below 0.1, and then intersected the two groups to plot a Venn diagram to show the unique and common lncRNAs between NOR and DOR (Fig. 2c). To further probe the functions of these lncRNAs, we forecasted the cis-regulated target genes of the DE lncRNAs between the DOR group and NOR group. A total of 52 lncRNAs were found to have target genes if 100 kilobase pairs (kbp) was used as the cutoff. Some lncRNAs had 2–3 target genes, and 57 probable lncRNA target genes were identified (Supplemental Table 4). Based on these cis-regulated target genes, analyses of functional enrichment were performed using the Gene Ontology (GO) (http://geneontology.org/) database. A total of 76 GO terms were enriched significantly (*P* < 0.05). For biological process (BP), 57 terms were found, including “stem cell proliferation (GO:0072089). For “cellular component” (CC), 6 terms were found, including “proteasome activator complex” (GO:0008537). For molecular function (MF), 13 terms were found, including “CXCR3 chemokine receptor binding” (GO:0048248) (Fig. 2d-e).

We used the Kyoto Encyclopedia of Genes and Genomes (KEGG) (www.genome.jp/kegg/) database to identify signaling pathways significantly enriched for lncRNA target genes. Three significantly enriched signaling pathways related to the lncRNA target genes (*P* < 0.05) were found, including “ferroptosis”, “fatty acid biosynthesis”, and the “JAK-STAT signaling pathway” (Fig. 2f).

We discovered the predicted outcomes of the DE lncRNAs with cis-regulated genes. We listed the first-six and last-four lncRNA–gene pairs according to the Pearson correlation coefficient. The first-six lncRNA–gene pairs were regulated in the same direction, whereas the last-four lncRNA–gene pairs were in the opposite direction (Table 3).

**Table 3:** Differentially expressed lncRNA–gene pairs between the DOR group and NOR group

Gene	lncRNA transcript	***Cis*** location (bp)	Pearson correlation coefficient
CXCL10	MSTRG.57192.1	1 K	1.00
TNFSF15	MSTRG.77544.1	100 K	1.00
PGBD5	MSTRG.9312.2	100 K	1.00
CNTN3	MSTRG.52583.1	100 K	0.99
SLC16A10	MSTRG.66173.1	10 K	0.99
SLC16A10	MSTRG.66167.1	100 K	0.99
BNIP2	MSTRG.27592.2	100 K	−0.31
SYNCRIP	ENST00000656092	100 K	−0.32
NPIPB15	MSTRG.31257.1	10 K	−0.33
C2CD2	MSTRG.48668.1	100 K	−0.35

### Expression profile of mRNAs

**Fig. 3:**
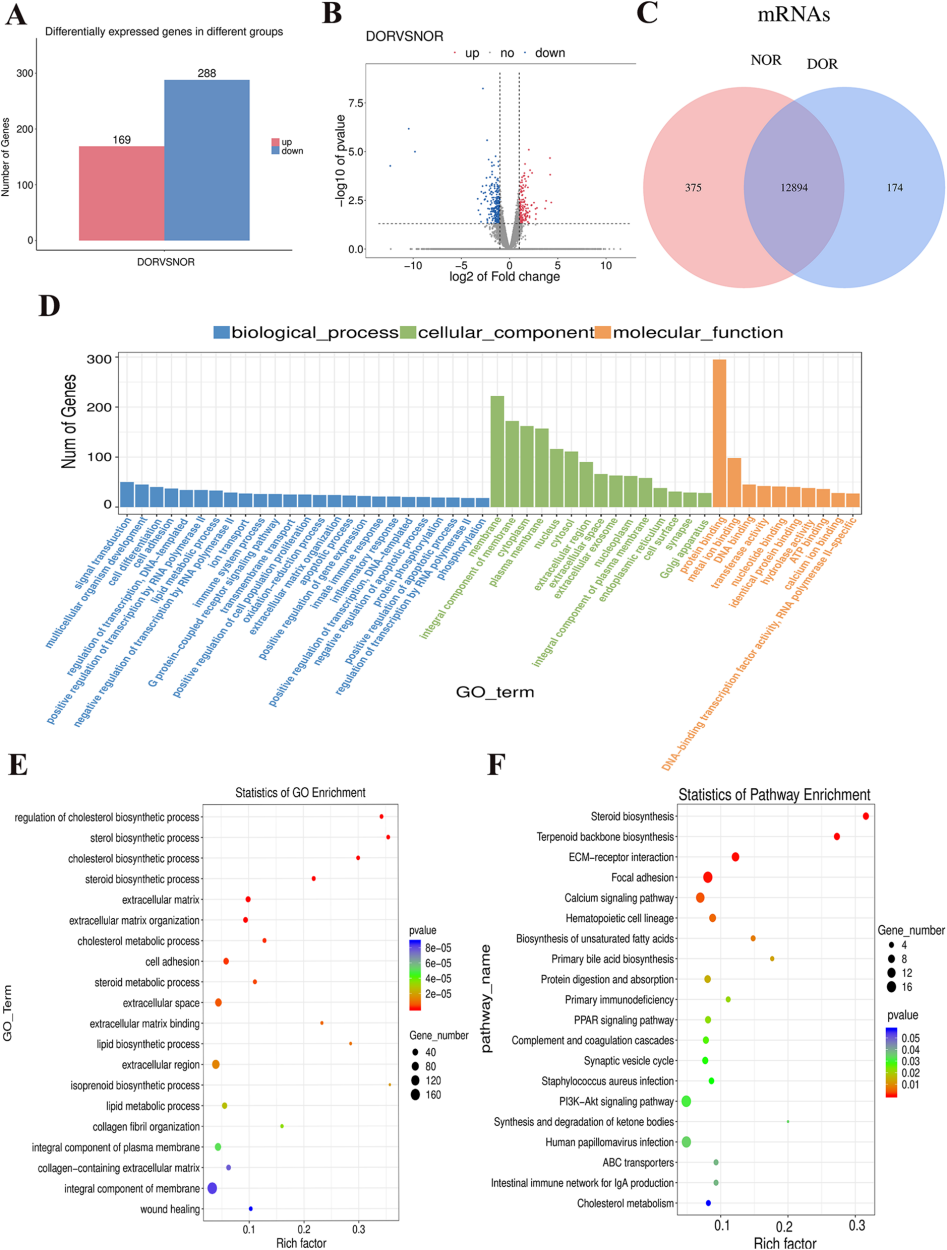
Identification and characterization of the DE mRNA genes between women in the DOR group and NOR group. **a** Number of DE mRNA genes showing upregulated and downregulated expression. **b** Volcano plot of the DE mRNA genes. **c** Venn diagram showing the number of unique and common mRNAs between the two groups. **d** Histogram of the GO-enriched terms of DE mRNA genes. **e** Scatter plot of the GO-enriched DE mRNA genes. **f** Scatter plot of the signaling pathway enrichment of DE mRNA genes using the KEGG database

A total of 457 DE mRNA genes were found between the OGCs of the two groups. Of these genes, 169 genes were upregulated, and 288 genes were downregulated (Fig. 3a-b). We similarly plotted Venn diagram to demonstrate the unique and common mRNAs between NOR and DOR (Fig. 3c). To further explore the functions of these DE mRNA genes, analyses of functional enrichment were performed using the GO database. Among the 457 DE mRNA genes, 3404 GO terms with functional information were enriched (Fig. 3d-e). A total of 542 GO terms were enriched significantly: 359 BP terms (e.g., ‘cell adhesion’, GO:0007155; ‘positive regulation of apoptotic process’, GO:0043065 and ‘steroid biosynthetic process’, GO:0006694), 137 MF terms (e.g., ‘oxidoreductase activity’, GO:0016491), and 42 CC terms (e.g., ‘endoplasmic reticulum lumen’, GO:0005788) (Supplemental Table 5). Analyses of signaling pathway enrichment using the KEGG database revealed that the DE mRNA genes were significantly enriched in nineteen signaling pathways, including “steroid biosynthesis”, “focal adhesion”, “ECM–receptor interaction”, “PPAR signaling pathway”, and “PI3K-AKT signaling pathway” (Fig. 3f).

### Coenriched GO terms of the DE lncRNAs and mRNAs

We then aimed to identify the key pathways that regulate the ovarian reserve. Hence, we identified five significantly enriched GO terms in the enrichment of the DE mRNAs and the enrichment of the lncRNA target genes. The significantly enriched GO terms were “CXCR chemokine receptor binding”, “CXCR3 chemokine receptor binding”, “positive regulation of transforming growth factor-beta production”, “regulation of bile acid biosynthetic processes”, and “cellular response to organonitrogen compound”. Three pathways were involved in BP. and the other two pathways were involved in MF (Table 4).

**Table 4:** Coenriched GO terms of DE lncRNAs and mRNAs

GO Term	GO function	P
GO:0048248 (CXCR3 chemokine receptor binding)	molecular_function	0.002
GO:0071636 (positive regulation of transforming growth factor beta production)	biological_process	0.005
GO:0045236 (CXCR chemokine receptor binding)	molecular_function	0.010
GO:0070857 (regulation of bile acid biosynthetic process)	biological_process	0.027
GO:0071417 (cellular response to organonitrogen compound)	biological_process	0.034

### Construction of a co-expression network of DE lncRNAs and mRNAs

**Fig. 4:**
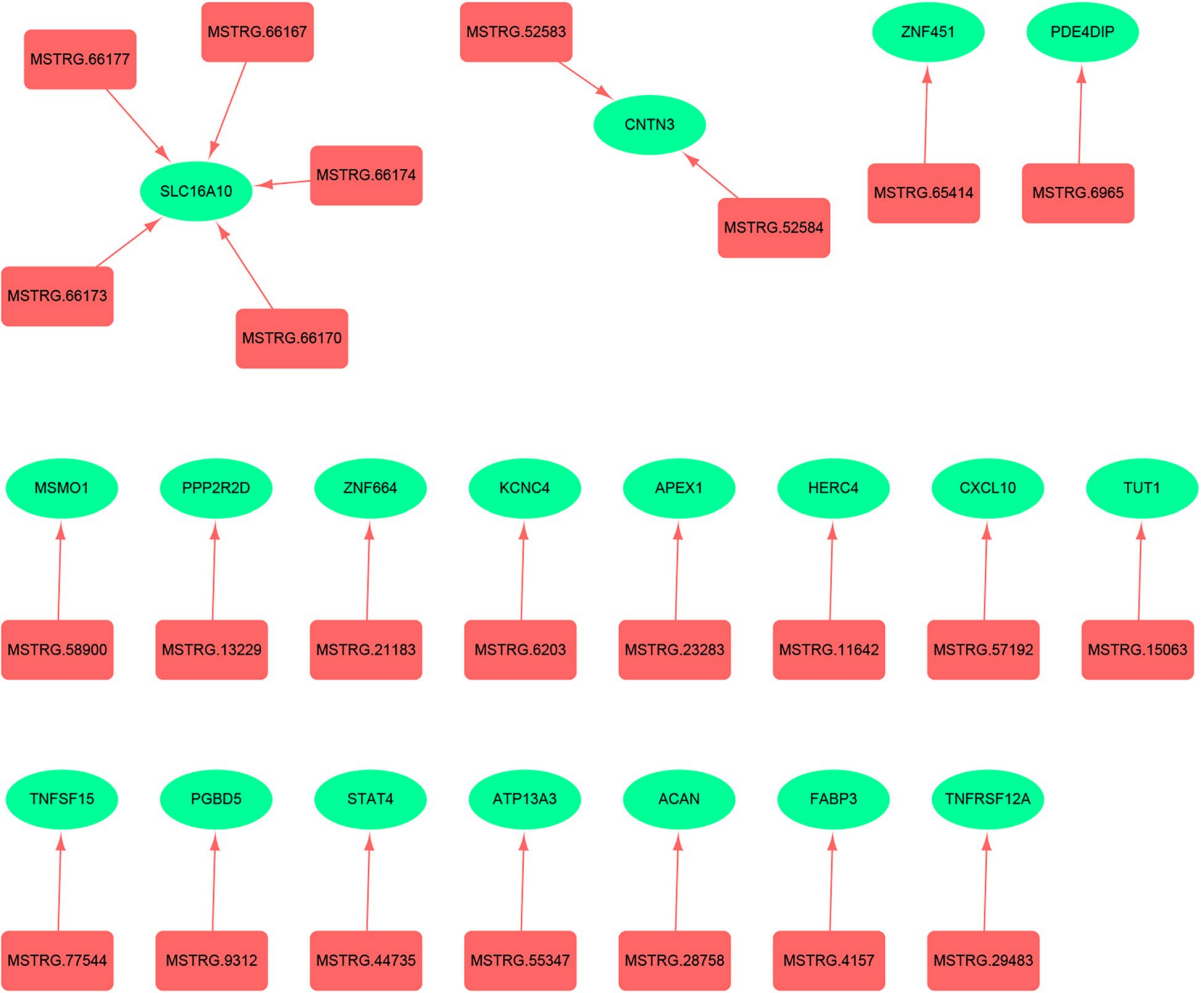
The lncRNA–mRNA regulatory network. Each lncRNA is a red round rectangle. Each mRNA is a green ellipse

To further explore the potential regulatory mechanisms, we constructed lncRNA-mRNA co-expression networks and performed comprehensive analyses. After cis-regulated target genes were predicted for DE lncRNAs, 57 target genes were identified that were cis-regulated by 52 lncRNAs, both of which were DE between DOR and NOR groups. Twenty-four positively correlated lncRNA–mRNA co-expression pairs containing 24 DE lncRNAs and 19 DE mRNAs were acquired at the verge of a Pearson correlation coefficient (r > 0.40) (Supplemental Table 6). Then, a lncRNA–mRNA regulatory network comprising dysregulated lncRNAs and their cis-target mRNAs was visualized using Cytoscape v3.7.1 (https://cytoscape.org/). Solute carrier family 16 member 10 (SLC16A10) was included in this network and was regulated by five lncRNAs: MSTRG.66173, MSTRG.66167, MSTRG.66177, MSTRG.66174, and MSTRG.66170 (Fig. 4). Among them, SLC16A10 was enriched in several GO terms, including “protein binding” (GO:0005515), “thyroid hormone generation” (GO:0006590), and “cell junction” (GO:0030054). SLC16A10 was also enriched in the “thyroid hormone signaling pathway” and “protein digestion and absorption”.

### Verification of the DE lncRNAs and mRNAs by RT–qPCR

To verify the RNA-seq results, RT–qPCR was performed on the seven DE lncRNAs and mRNAs respectively identified between the OGCs from the DOR group and NOR group. Table 5 displayed the baseline characteristics of the case used for RT-qPCR validation, with patients in the DOR group not significantly different from those in the NOR group (*P* > 0.05), except for indicators related to ovarian reserve such as AMH level, AFC, and number of oocytes retrieved (*P* < 0.05). These seven lncRNAs include nuclear rich abundant transcript 1 (NEAT1), Ras-related protein in brain 5A (RAB5A), maternally expressed gene 3 (MEG3), guanine nucleotide-binding protein subunit gamma-12 (GNG12), macrophage stimulating 1 like (MST1L), general control nonderepressible 1 (GCN1), zinc-finger E-box binding homeobox 2-AS1(ZEB2-AS1). These seven mRNAs include fibronectin 1 (FN1), hyaluronan synthase 3 (HAS3), matrix metalloproteinase 15 (MMP15), glyoxalase 1 (GLO1), regulator of G-protein signaling 4 (RGS4), sulfite oxidase (SUOX), Semaphorin 3A (SEMA3A). Table 6 presented information on the relevant sequencing results for the above lncRNAs and mRNAs.

**Table 5:** Comparison of the baseline characteristics between the NOR group and DOR group used for RT-qPCR

	NOR (***n*** = 6)	DOR (n = 6)	P
Patient age (years)	30.50 ± 2.88	35.50 ± 4.72	0.057
Infertility duration (years)	3.00 ± 1.67	3.50 ± 2.43	0.687
Infertility type (%)			1.000
Primary	2/6 (33.3)	1/6 (16.7)	
Secondary	4/6 (66.7)	5/6 (83.3)	
BMI (kg/m^2^)	20.20 ± 2.61	21.52 ± 2.86	0.424
AMH (ng/mL)	4.13 ± 0.59	0.78 ± 0.22	< 0.001
Basal FSH (mIU/mL)	6.41 ± 1.48	11.02 ± 4.42	0.051
AFC (n)	26.00 ± 2.68	7.00 ± 2.10	< 0.001
Previous IVF/ICSI attempts (n)	0.67 ± 1.21	0.67 ± 0.82	1.000
Number of oocytes retrieved	16.50 ± 7.79	4.17 ± 1.84	0.011

Data are the mean ± SD and proportion (%)

*AMH* Anti-Müllerian hormone, *AFC* Antral follicle count, *BMI* Body mass index, *FSH* Follicle-stimulating hormone, *ICSI* Intracytoplasmic sperm injection, *IVF* In vitro fertilization

**Table 6:** Sequencing information of lncRNAs and mRNAs used for RT-qPCR verification

	Transcript name	Gene name	log_**2**_(fold_change)	Regulation	P
lncRNAs	MSTRG.15237.9	NEAT1	−4.69	Down	0.041834004
	MSTRG.50996.10	RAB5A	−3.07	Down	0.003518952
	MSTRG.25915	MEG3	−3.83	Down	0.176775112
	MSTRG.5280.1	GNG12	−4.85	Down	0.000398357
	MSTRG.3657.2	MST1L	−4.88	Down	0.000306066
	MSTRG.20933.1	GCN1	5.98	Up	0.000182295
	MSTRG.43755	ZEB2-AS1	−13.50	Down	0.000166045
mRNAs	MSTRG.45478	FN1	−1.12	Down	0.003516700
	MSTRG.31094	HAS3	1.60	Up	0.000043116
	MSTRG.30842	MMP15	1.11	Up	0.008575602
	MSTRG.64747	GLO1	−1.84	Down	0.000036052
	MSTRG.7545	RGS4	−2.17	Down	0.000025747
	MSTRG.19257	SUOX	1.29	Up	0.000206956
	MSTRG.70183	SEMA3A	−1.16	Down	0.000032761

**Fig. 5:**
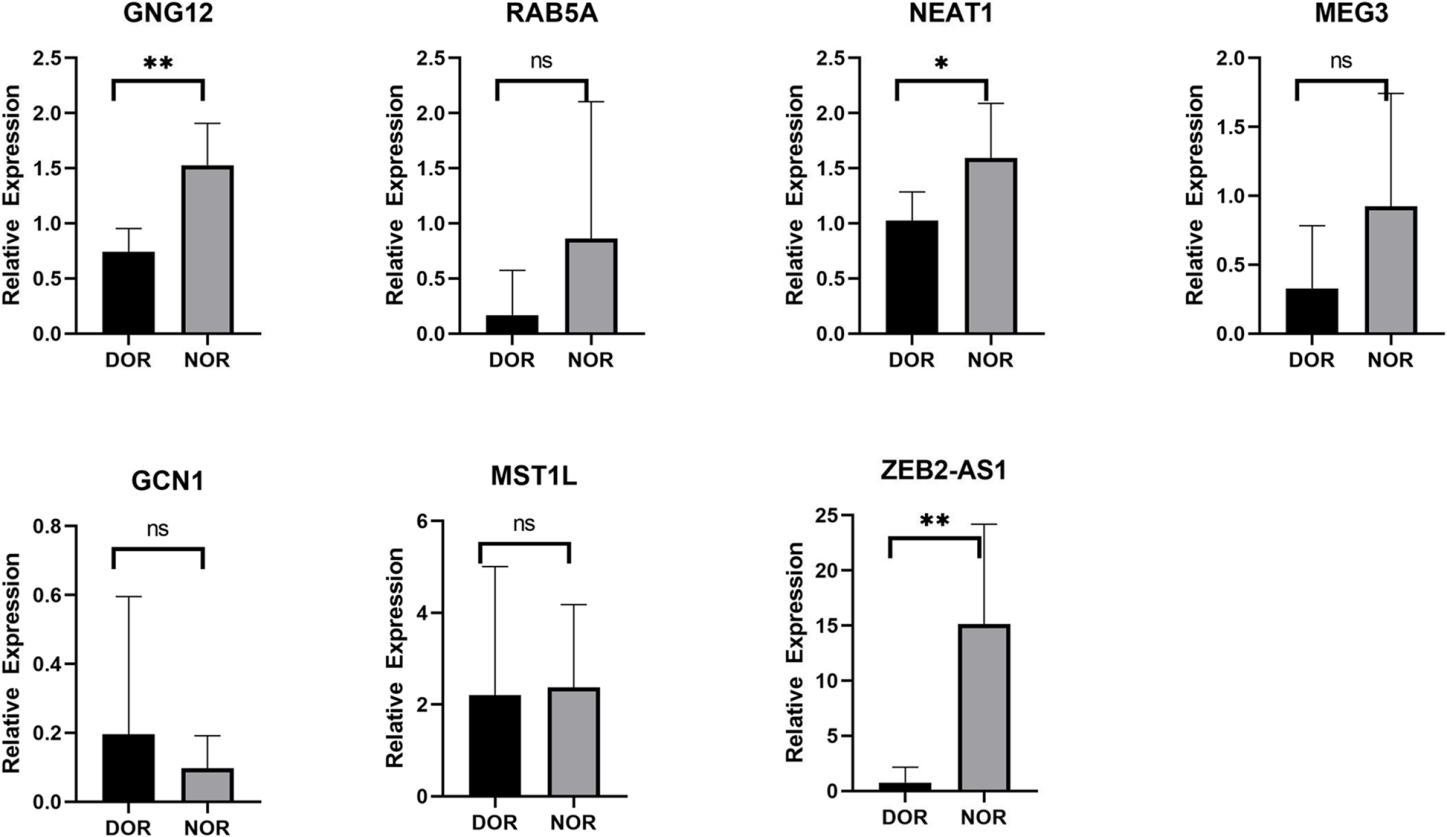
Expression of key lncRNAs as determined by RT–qPCR. ns, *P* > 0.05; *P < 0.05; **P < 0.01

**Fig. 6:**
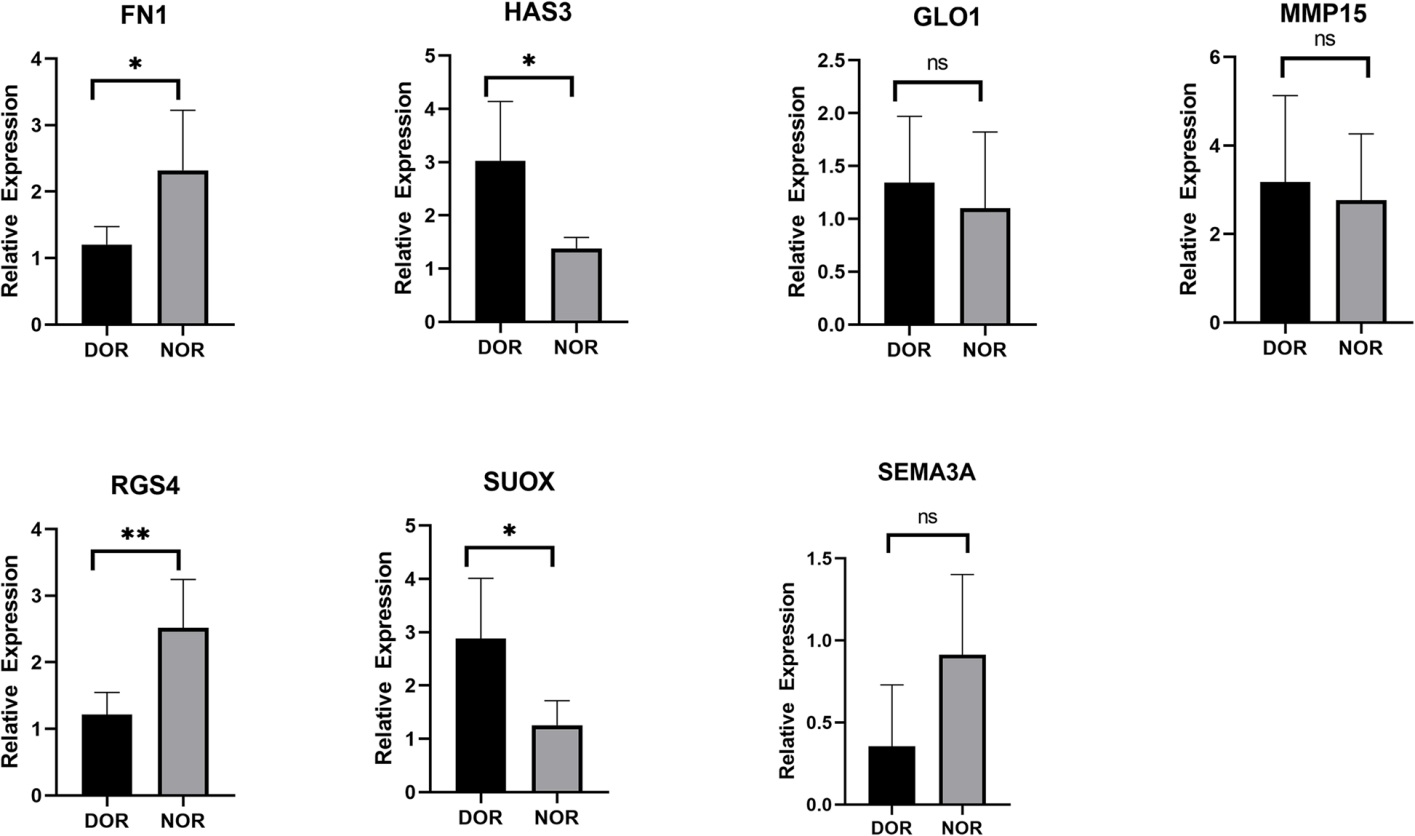
Expression of key mRNAs as determined by RT–qPCR. ns, *P* > 0.05; *P < 0.05; **P < 0.01

Among the lncRNAs, the expression of NEAT1, GNG12, and ZEB2-AS1 was consistent with the RNA-seq results. The expression of the remaining 4 lncRNAs was not significantly different (Fig. 5). Among the mRNAs, the expression of FN1, HAS3, RGS4, and SUOX was consistent with the RNA-seq results. The expression of the remaining 3 mRNAs did not differ significantly (Fig. 6). These results indicate that the seq-RNA data were reliable.

## Discussion

Follicular reserve and ovarian function are measures of reproductive endocrine functionality and fertility potential [15]. OGCs play important roles in the regulation of follicle activation, growth, development, and atresia [16]. Recently, several studies targeting specific genes and regulating various signaling pathways have shown that lncRNAs have vital roles in the formation and development of ovarian follicles [17–19].

We report, for the first time, the expression profiles of lncRNAs and mRNAs in OGCs of DOR patients and NOR patients, as identified by high-throughput sequencing. We identified 466 DE lncRNAs and 457 mRNAs associated with ovarian reserve. We showed that these dysregulated genes were involved in multiple biological processes, including cell adhesion and apoptosis, the immune system, and signaling pathways such as steroid biosynthesis. Notably, multiple lncRNAs could contribute to DOR by targeting and regulating signaling pathways mediated by SLC16A10, such as thyroid hormone synthesis and protein digestion and absorption. Overall, our results indicate the potential regulatory role of related lncRNAs and mRNAs in DOR.

In recent years, several studies have identified an important role for lncRNAs in the regulation of follicle development [20, 21]. Studies have identified differential expression of lncRNAs associated with reproductive function by using RNA-seq of OGCs obtained from humans [22], with most studies focusing on PCOS [23], endometriosis [24, 25], and POF [26, 27]. The lncRNAs we identified had fewer exons, shorter ORFs, and shorter transcript lengths compared with those in protein-coding transcripts, and these characteristics were similar to those from porcine OGCs [28] and human placentas at term [29]. These data demonstrate that the lncRNA outcomes we obtained were reliable.

Several research teams have reported aberrant lncRNA expression in POI and identified hundreds of lncRNAs associated with POI. Some of these lncRNAs could be used as biomarkers for the diagnosis or prognosis of DOR [18, 30]. The discrepancies between the DE lncRNAs identified in our study and those in other studies are due (at least in part) to differences in sample selection and sequencing methods. Moreover, several researchers have conducted functional studies on these DE lncRNAs [31–33] and reported different underlying molecular mechanisms. However, most of those studies concentrated on a particular gene or signaling pathway and did not analyze them from a holistic perspective. Additionally, most of the identified lncRNAs and mRNAs were associated with proliferative and apoptotic processes. The DE genes identified in our study were mostly involved in the signaling pathways related to the adhesion, proliferation, and apoptosis of cells, steroid biosynthesis and metabolism, and the immune response. All of these are biological processes involved in follicular atresia induced by OGC apoptosis [34, 35].

Folliculogenesis is regulated by multiple mechanisms involving endocrine and intraovarian signaling pathways [36, 37], and changes caused by the differential expression of genes may further affect ovarian reserve. We revealed several DE lncRNAs and mRNAs that could be associated with ovarian reserve. The lncRNAs validated by RT-qPCR were NEAT1, GNG12, and ZEB2-AS1; the mRNAs were FN1, HAS3, RGS4, and SUOX, and we will focus on the above genes. NEAT1 is a relatively well-studied lncRNA involved in the formation and maintenance of a “nuclear paraspeckle” (a nuclear body with multiple gene-expression functions). NEAT1 can promote disease through regulation of mitochondrial function [38]. NEAT1 is expressed abundantly in oocytes as well as in primordial, primary, and small antral follicles. It is involved mainly in the expression of genes related to apoptosis and extracellular matrix-related functions to achieve epigenetic control of follicular development [39, 40]. It was found that NEAT1 was DE in patients with PCOS [41] and POF [42], and that overexpression or interference with NEAT1 could improve the pathological changes in rat ovarian tissue by affecting the apoptosis of OGCs. GNG12, a particular G protein-coupled receptor, which plays crucial roles in the proliferation of tumor cells as a transducer and transmembrane signaling regulator. ZEB2-AS1 [43, 44] and GNG12 [45] can regulate cell proliferation, apoptosis, and migration processes and thus promote tumor cell growth of by affecting various pathways such as phosphoinositide 3-kinase/protein kinase B (PI3K/AKT), Wnt/β-catenin, etc. However, they have not been adequately investigated in the context of follicle growth, and the lonely study has confirmed that ZEB2-AS1 could enhance the activity of trophoblast cells and prevents the development of recurrent spontaneous abortion [46]. In the present study, we observed lncRNA GNG12 and ZEB2-AS1 expression was downregulated in the OGCs from DOR patients, suggesting it may contribute to DOR development by increasing apoptosis and inhibiting cell proliferation in OGCs.

Among the DE mRNAs, FN1 is a glycoprotein component of the extracellular matrix that is widely involved in processes such as cell migration, adhesion, and proliferation. FN1 was found to be low in PCOS patients with decreased angiogenic capacity [47]. Combined with the finding in this study that FN1 expression was decreased in women with DOR, we hypothesized that compromised vascularization around the ovarian follicles may lead to follicular growth arrest. SUOX regulated sulfite oxidase converts sulfite to sulfate in vivo, which has been found to be associated with PCOS [48] and the GO term “mitochondrion” [49]. Our study found low expression of SUOX in DOR patients, which may contribute to the development of DOR by affecting mitochondrial metabolic pathways. As for HAS3, its regulated synthesis of hyaluronan has the property of maintaining tissue homeostasis. A study has shown that there was a lower level of HAS3 in PCOS endometrium compared to women with regular menstrual cycles in the proliferative phase [50]. Similar to the findings of our study, when goat oocytes were cultured in vitro, the levels of HAS3 showed a likewise upward trend as the oocytes aged, and the number of apoptotic cells increased [51]. The variability in the above findings may be due (at least in part) to differences in species, sample selection and disease. This also suggests that HAS3 plays an important role in reproductive disorders, but the exact mechanism of action is unclear. We look forward to further studies on the role of HAS3 in influencing follicle development and oocyte quality in the future. RGS4, a GTPase-activating protein, changes in its expression levels can affect apoptosis, invasion and migration capacity and is linked to the development of diseases such as cancer [52]. It has been less studied in the reproductive system, where it has been found to accelerate the kinetics of K(+) channels in the Xenopus oocyte system [53]. This study found that RGS4 expression was down-regulated in DOR patients, this result provides a direction for future research, the exact action mechanism still needs to be further clarified.

We assessed the enrichment of functions and signaling pathways using the GO database and KEGG database, respectively, to reveal the biological functions of the DE lncRNA target genes and mRNAs. The DE genes were involved mainly in cell adhesion, steroid biosynthesis, and pathways related to immunity and apoptosis, which are the functional pathways of OGCs [34, 54, 55]. Cell adhesion is involved in the in vitro culture of porcine [56] and human [57] OGCs and can affect the proliferative potential and survival capability. Pashaiasl and collaborators measured the mRNA expression profile of the OGCs of DOR patients. Similar to our findings, they identified DE genes involved in focal adhesion [58], as well as DE genes involved in ossification, ovarian-follicle development, vasculogenesis, and sequence-specific activity of DNA-binding transcription factors. OGCs are the most important somatic cells for the synthesis of steroid hormones. We found that the DE genes could affect steroid biosynthesis and thus regulate the proliferation and apoptosis of OGCs through multiple pathways. These findings have been documented in studies in sheep [59], mice [60] and women with POF [61].

Autoimmunity is responsible for approximately 4–30% of POI cases [62]. Women suffering from POI carry a high risk of having autoimmune disorders [63]. Chemokines play an important role in reproductive immunology [64] and regulate leukocyte migration by attracting cells that express their cognate ligands. C-X-C motif chemokine ligand 12 (CXCL12), C-X-C motif chemokine receptor 4 (CXCR4), and CXCR7 can influence the development of endometriosis, Asherman’s syndrome, endometrial cancer, and ovarian cancer [65]. CXCR3, which was identified in the present study, has been investigated less thoroughly. Therefore, women should be screened for common autoantibodies (e.g., steroid cell, anti-ovarian, and anti-thyroid autoantibodies), which can aid in the early diagnosis and treatment of DOR and the prevention of POF. A prospective cohort study involving 164 women found a higher prevalence of OGC apoptosis, worse ovarian response, lower oocyte count, lower embryo count, and poor pregnancy outcomes in patients with DOR [66].

We found that the PI3K-AKT and Janus kinase/signal transducer and activator of transcription (JAK-STAT) pathways enriched by the DE genes were associated with apoptotic processes. Several studies have demonstrated that the inhibition of the PI3K-AKT pathway leads to apoptosis and autophagy in OGCs [60, 61]. The importance of the JAK/STAT signaling pathway in ovarian development and folliculogenesis in horses [67] and humans [68] has been demonstrated. Polyfluoroalkyl substances reduce the ovarian reserve and decrease endogenous hormone synthesis by activating the peroxisome proliferator-activated receptor (PPAR) pathway [69], which has been indicated as a new biomarker of follicular capacity [70]. By constructing a lncRNA–mRNA co-expression network, we found that SLC16A10 was positively regulated by multiple lncRNAs. Additionally, the function of the target gene SLC16A10 was enriched mainly in thyroid hormone synthesis as well as the binding, digestion, and absorption processes of proteins. One retrospective study found that increased levels of thyrotropic hormone may be associated with decreased serum levels of AMH [71], whereas the ovarian reserve was not associated with anti-thyroid peroxidase antibodies or anti-thyroglobulin antibodies. Transplantation of human amniotic epithelial cells has been shown to restore ovarian function and improve ovarian reserve and fertility in cyclophosphamide-induced POI rats by affecting protein digestion/absorption, and steroid-biosynthesis pathways [72].

## Conclusions

The expression profiles of lncRNAs and mRNAs in OGCs from women with DOR and NOR were mined using transcriptome sequencing. A total of 466 lncRNAs and 457 mRNAs were identified to have differential expression in the transcripts. Bioinformatic analysis revealed that most of these lncRNAs and mRNAs were involved in important pathways and biological processes related to cell adhesion, apoptosis, steroid biosynthesis, and the immune system. Dysregulated lncRNAs and mRNAs could serve as diagnostic biomarkers. Further studies should concentrate on the elucidation of specific molecular mechanisms to provide a new understanding for the diagnosis and treatment of DOR.

## Methods

### Ethical approval of the study protocol

The research protocol was approved by the Reproductive Medicine Ethics Committee of the Affiliated Hospital of Shandong University of Traditional Chinese Medicine (Ref: SZ2018090103). All patients provided written informed consent before sample collection.

### Patients and sample collection

Samples and clinical data were collected at the Center for Integrative Reproduction and Genetics, Affiliated Hospital of Shandong University of Traditional Chinese Medicine, from September 2018 to October 2020. Twenty-four patients were recruited in our study. These patients underwent IVF/ICSI using a gonadotrophin-releasing hormone antagonist for controlled ovarian hyperstimulation. All patients satisfied the following criteria: age of 25–40 years; body mass index (BMI) of 18–25 kg/m^2^; and no endocrine disease, uterine anomaly, endometriosis, or chromosomal abnormalities.

The study group (*n* = 12) comprised infertile women with DOR. The diagnostic criteria adopted an amended version of the Bologna standard [73], with participants fulfilling a minimum of two of the following conditions: (i) bilateral AFC < 6; (ii) AMH < 1.10 ng/mL; (iii) menstrual basal FSH level of 10–40 mIU/mL. The control group (*n* = 12) was women with NOR and pure male factor (azoospermia or severe oligospermia/aspermia) infertility. Six patient samples from each group were selected randomly for lncRNA sequencing. The other six samples underwent RT–qPCR.

Oocyte retrieval was performed 36 h after injection of human chorionic gonadotropin once follicles > 17 mm in diameter. Follicular fluid was obtained from the first aspirated follicle of each ovary during ultrasound-guided transvaginal ovarian puncture for oocyte retrieval. OGCs were collected and purified from the follicular fluid by density gradient centrifugation. Follicular fluid was centrifuged at 380×*g* for 5 min at 23 °C and the supernatant was removed. Phosphate-buffered saline (5 mL) was added to the precipitate and mixed. Then, 5 mL of Ficoll-Paque (GE Healthcare, Chicago, IL, USA) was added to a 15-mL centrifuge tube. The suspension was added slowly to the upper layer of Ficoll-Paque, and centrifugation at 380×*g* for 20 min at 23 °C was carried out. The OGC layer was aspirated and transferred to a 1.5-mL centrifuge tube, and repeated pipetting was undertaken followed by centrifugation at 380×*g* for 3 min at 23 °C. Finally, the supernatant was removed and the OGCs were stored in a − 80 °C for further analyses.

### Preparation and sequencing of RNA libraries

TRIzol® Reagent (Invitrogen, Carlsbad, CA, USA) was employed to isolate and purify total RNA according to the manufacturer’s instructions. The amount and purity of total RNA in each sample was analyzed using a bioanalyzer (2100 series; Agilent Technologies, Santa Clara, CA, USA) and an RNA Nano1000 Assay Kit (Agilent Technologies) so that the RNA integrity number (RIN) > 7.0. Ribosomal RNA was removed according to the instructions of the Ribo-Zero™ rRNA Removal Kit (Illumina). At 95 °C, the remaining RNA was broken into short fragments using divalent cations. Subsequently, cDNA was created by reverse transcription using fragmented RNA as a template, followed by addition of deoxynucleoside triphosphate, RNase H, and *Escherichia coli* DNA polymerase I to synthesize the second strand. After purification of AMPure XP beads (Beckman Coulton, Fullerton, CA, USA) and end repair, poly(A) was added, and sequencing connectors were attached. Then, cDNA of a certain length range was extracted. PCR amplification was performed to obtain a cDNA library. The average insert size of the final cDNA library was 300 ± 50 bp. Twelve separate RNA-seq libraries were generated for the study group and control group.

### RNA-seq

High-throughput sequencing of samples was executed on a NovaSeq 6000 using 2 × 150-bp paired-end sequences (PE150) according to a standard procedure. First, reads containing adapter contamination, low-quality bases, or unidentified bases were excluded by applying cutadapt v1.9 [74] (https://cutadapt.readthedocs.io/en/stable/#). Then, the quality of reads was authenticated using FastQC v0.10.1 [75] (www.bioinformatics.babraham.ac.uk/projects/fastqc/). The Q20, Q30, and GC contents of clean data were calculated, and subsequent analyses were based on these data. Sequencing and data acquisition were undertaken by LC-Bio Technology (Hangzhou, China).

### Transcript assembly

HISAT (v2.0.4) [76] was utilized to preprocess raw data and map the processed valid data to the human reference genome (GRCh38). Annotation files in Gene Transfer Format were created to identify which genes these reads mapped to. The mapped reads from each sample were assembled using StringTie v1.3.0 [77] (https://ccb.jhu.edu/software/stringtie/index.shtml/) with default parameters. Then, all transcripts in the sample were joined using gffcompare (https://ccb.jhu.edu/software/stringtie/gffcompare.shtml) to build a whole transcriptome. Then, StringTie was used to calculate the FPKM to quantify the expression of the transcripts.

### lncRNA identification

First, transcripts that overlapped with known mRNAs and transcripts shorter than 200 bp were removed. If the remaining transcripts duplicated the lncRNA, then these transcripts were deemed to be “known lncRNAs”. The rest of the non-annotated transcripts were assumed to recognize the underlying novel lncRNAs. Then, we employed CPC (0.9-r2) [78] and CNCI (v2.0) [79] to forecast the coding potential of the remaining transcripts of length ≥ 200 bp, mapped read coverage ≥3, and exon number ≥ 1. Transcripts with a CPC score < − 1 and CNCI score < 0 were assumed to be potential novel lncRNAs.

### Differential expression of mRNAs and lncRNAs

StringTie was adopted to evaluate the expression of mRNAs and lncRNAs by calculation of FPKM. Multiple values were analyzed by the packages -edgeR [80] or DESeq2 [77] within R (R Institute for Statistical Computing, Vienna, Austria). *P* < 0.05 and | log2 (fold change) | ≥1 were used as the basis for screening DE mRNAs and lncRNAs between the DOR group and NOR group.

### Target-gene prediction and functional analyses of lncRNAs

The interaction between lncRNAs and their adjacent genes is known as “*cis*-regulation”. We used Python scripts to predict the *cis*-regulatory relationships between mRNAs and lncRNAs in the 100-kbp range upstream and downstream of chromosomes. We undertook analyses of enrichment of the functions and signaling pathways of lncRNA-targeted mRNAs using GO and KEGG databases [81, 82], respectively.

### Validation of lncRNA expression by RT–qPCR

Based on the analysis of sequencing results, literature review and research needs, the expression of seven lncRNAs (GNG12, MEG3, NEAT1, MST1L, RAB5A, GCN1 and ZEB2-AS1) and mRNAs (HAS3, MMP15, FN1, GLO1, RGS4, SUOX, SEMA3A) was measured by RT–qPCR in OGC samples from 12 patients to validate the RNA-seq results. Total RNA was extracted from samples using the RNeasy Micro Kit following the manufacturer’s instructions (Qiagen, Stanford, VA, USA). The content and purity of RNA were measured at an absorbance of 260/280 nm using Scandrop™ 100 (Analytik Jena, Jena, Germany). Total RNA was reverse-transcribed into cDNA using the TUREscript First Strand cDNA Synthesis Kit (Aidlab, Beijing, China) according to the manufacturer’s instructions. The specific primers designed for RT–qPCR using Primer-Blast (National Center for Biotechnology Information) and primer-primer6 together are shown in Supplemental Table 7. The analytikjena-qTOWER2.2 PCR System (Analytik Jena) was applied to conduct RT–qPCR. The reaction protocol was as follows: initial denaturation at 95 °C for 3 min, denaturing at 95 °C for 10 s, and annealing at 60 °C for 30 s for 40 cycles. Glyceraldehyde − 3-phosphate dehydrogenase, a housekeeping gene, was selected as a reference gene because of its relatively stable and high expression in ovarian granulosa cells [83]. The relative expression levels of lncRNAs and mRNAs were calculated by the 2^−ΔΔCt^ method [84]. For each reaction, three independent biological replicates were employed.

### Statistical analyses

SPSS 23.0 (IBM, Armonk, NY, USA) was used for statistical analyses. Prism 8.0.1 (GraphPad, San Diego, CA, USA) was employed for statistical analyses of RT–qPCR results and graphs. The Shapiro-Wilk test was used to assess the normality of the data distribution. Measurement data were expressed as the mean ± SD. Continuous variables were compared using the Student’s t-test or Welch’s t-test. As for categorical variables, the differences between groups were analyzed using the Chi-square test (χ^2^). *P* < 0.05 was deemed significant.

## Supplementary Information

**Additional file 1 Supplemental Table 1** Alignment of the sequencing reads to the reference genome. **Supplemental Table 2** Statistical analyses of lncRNAs in each sample. **Supplemental Table 3** Statistical analyses of the DE lncRNAs. **Supplemental Table 4** Statistical analyses of the DE lncRNA *cis*-regulated target genes. **Supplemental Table 5** GO terms enriched for the DE mRNA genes. **Supplemental Table 6** The lncRNA–mRNA coexpression pairs. **Supplemental Table 7** Specific primer sequences of the validated mRNAs and lncRNAs.

## Data Availability

All the raw data has been stored in GEO (Gene Expression Omnibus) with the login number GSE193136 (https://www.ncbi.nlm.nih.gov/ geo/query/acc.cgi?acc = GSE193136).

## Abbreviations

AFC: Antral follicle count
AMH: Anti- Müllerian hormone
BMI: Body Mass Index
BP: Biological process
CC: Cellular component
cDNA: Complementary DNA
CNCI: Coding-Non-Coding Index
CPC: Coding Potential Calculator
DE: Differentially expressed
DOR: Diminished ovarian reserve
FPKM: Fragments per kilobase of exon model per million mapped reads
FSH: Follicle-stimulating hormone
GO: Gene Ontology
ICSI: Intracytoplasmic sperm injection
IVF: In vitro fertilization
KEGG: Kyoto Encyclopedia of Genes and Genomes
lncRNAs: Long noncoding RNAs
MF: Molecular function
NOR: Normal ovarian reserve
OGCs: Ovarian granulosa cells
ORF: Open reading frame
PCOS: Polycystic ovary syndrome
POF: Premature ovarian failure
POI: Premature ovarian insufficiency
PPAR: Peroxisome proliferator-activated receptor
RT–qPCR: Reverse transcription-quantitative polymerase chain reaction
RNA-seq: RNA-sequencing
